# Midwest pharmacists’ familiarity, experience, and willingness to provide pre-exposure prophylaxis (PrEP) for HIV

**DOI:** 10.1371/journal.pone.0207372

**Published:** 2018-11-14

**Authors:** Jordan M. Broekhuis, Kimberly K. Scarsi, Harlan R. Sayles, Donald G. Klepser, Joshua P. Havens, Susan Swindells, Sara H. Bares

**Affiliations:** 1 College of Medicine, University of Nebraska Medical Center, Omaha, Nebraska, United States of America; 2 Department of Pharmacy Practice, University of Nebraska Medical Center, Omaha, Nebraska, United States of America; 3 Department of Biostatistics, University of Nebraska Medical Center, Omaha, Nebraska, United States of America; 4 Department of Internal Medicine, University of Nebraska Medical Center, Omaha, Nebraska, United States of America; Centers for Disease Control and Prevention, UNITED STATES

## Abstract

**Introduction:**

Pharmacist provision of pre-exposure prophylaxis (PrEP) through collaborative practice agreements with physicians could expand access to people at risk for HIV. We characterized pharmacists’ familiarity with and willingness to provide PrEP services in Nebraska and Iowa.

**Methods:**

An invitation to complete an 18-question survey was emailed to 1,140 pharmacists in Nebraska and Iowa in June and July of 2016. Descriptive analyses and Pearson chi-square tests were used to determine to what extent demographics, familiarity and experience were associated with respondent willingness to provide PrEP. Wilcoxon rank-sum tests compared ages and years of experience between groups of respondents.

**Results:**

One hundred forty pharmacists (12.3%) responded. Less than half were familiar with the use of PrEP (42%) or the CDC guidelines for its use (25%). Respondents who were older (p = .015) and in practice longer (p = .005) were less likely to be familiar with PrEP. Overall, 54% indicated they were fairly or very likely to provide PrEP services as part of a collaborative practice agreement and after additional training. While familiarity with PrEP use or guidelines did not affect respondents’ willingness to provide PrEP, respondents were more likely to provide PrEP with prior experience counseling HIV-infected patients on antiretroviral therapy (OR 2.43; p = 0.023) or PrEP (OR 4.67; p = 0.013), and with prior HIV-related continuing education (OR 2.77; p = 0.032).

**Conclusions:**

Pharmacist respondents in Nebraska and Iowa had limited familiarity and experience with PrEP, but most indicated willingness to provide PrEP through collaborative practice agreements after additional training. Provision of PrEP-focused continuing education may lead to increased willingness to participate in PrEP programs.

## Introduction

Despite evidence that pre-exposure prophylaxis (PrEP) using antiretrovirals can substantially reduce the risk of HIV infection in at-risk populations [1, 2] as well as CDC guidelines for its use [3], significant barriers exist to its prescription and use. Although recent data shows that PrEP use among commercially insured individuals in the United States increased from 3.3 per million in 2010 to 75.4 per million in 2014, there has not been as marked an increase in the North Central region of the country [4] With an estimated 47,165 new HIV diagnoses in the United States in 2015, prevention of HIV remains a critical public health issue [5, 6].

One of the key factors in successful implementation of HIV PrEP programs is finding providers who are willing to prescribe PrEP. In a qualitative study of barriers and facilitators to implementing PrEP, Krakower and colleagues noted that neither HIV specialists nor primary care providers (PCPs) considered PrEP implementation to fall within their clinical domain and have termed this the “purview paradox”[7]. Although HIV providers are most likely to come in contact with partners of persons living with HIV and are generally willing and able to fill this role, they do not have access to a large group of HIV-uninfected at-risk individuals and are not widely accessible, especially in rural areas [8]. PCPs often care for at-risk individuals but face many barriers to the provision of PrEP including time constraints, lack of training, lack of knowledge, discomfort with prescribing antiretrovirals, as wells as concerns about patient safety, adherence, and adverse effects [9]. This paradox is a major barrier to PrEP implementation in states like Nebraska and Iowa which contain many federally designated Medically Underserved Areas [10]. Access to PCPs in these regions can be challenging, and access to specialist physicians even more so.

Clinical pharmacists have a long history of participation in the care of persons with HIV, are increasingly involved in HIV prevention [11–13], and practice in a myriad of settings in which they interact with potential PrEP candidates. The community pharmacy, for example, has the potential to reach high volumes of individuals at risk for HIV. There are currently over 60,000 community pharmacies in the United States and there are 13 billion pharmacy visits each year in the United States which translates into an average of 530–570 visits per store each day [14].

State regulations govern the ability of pharmacists to provide patient care services through scope-of-practice laws and related rules [15]. In the United States, 48 states permit some type of pharmacist-prescriber collaborative practice, but not all states’ laws and regulations support the implementation of collaborative practice agreements (CPAs) which allow for the delegation of responsibilities from the collaborating prescriber to the pharmacist and thus enable the pharmacist to provide an array of patient care services [16]. Nebraska and Iowa both have laws explicitly authorizing CPAs, so pharmacists practicing in these states are able to participate in a variety of patient care services [15]. With the appropriate training, pharmacists could participate in a collaborative drug therapy management plan that would allow pharmacists working within the context of a defined HIV PrEP protocol to assume professional responsibility for all aspects of PrEP administration. Such a protocol would allow for increased access to PrEP services by simplifying the process and removing some of the barriers patients face when accessing PrEP.

What is not known, however, is Nebraska and Iowa pharmacists’ familiarity with and attitudes towards PrEP. Furthermore, although prior studies have assessed pharmacist knowledge and attitudes about HIV PrEP [17–19], they have not fully elucidated potential barriers to implementation of pharmacy-based PrEP models of care. The purpose of this study was to further characterize Nebraska and Iowa pharmacists’ PrEP-related familiarity and experience, to determine their willingness to implement PrEP services, and to identify some of the barriers to implementation of pharmacist-led PrEP services for patients at risk for HIV.

## Methods

### Study population

An e-mail invitation containing a link to the electronic survey was distributed to preceptors of pharmacy students in the College of Pharmacy at the University of Nebraska Medical Center and to pharmacists practicing in Nebraska and Iowa with contact information available through the Medical Monitoring Service, Inc.’s (MMS) database (www.mmslists.com). The MMS list is compiled from registrations, public records, state licensing boards and medical billing information for healthcare providers. All information is derived from proprietary, self-reported data, or sources of public record. It is obtained legally and ethically under strict list industry rules, regulations, and guidelines. The lists are updated quarterly by the National Change of Address process.

A single reminder e-mail was sent one week after the initial e-mail. Responses were collected from June 7 to July 27, 2016 A statement of informed consent was included at the beginning of the survey and no incentives were provided. The study was approved by the Institutional Review Board of the University of Nebraska Medical Center.

### Survey

An 18-question anonymous survey was developed and formatted with input from HIV providers, pharmacists, and a statistician (see S1 Appendix) and administered through Survey Monkey (www.surveymonkey.com). The survey was organized in five parts: demographics, experience, willingness, perceived abilities, patient-level barriers, and pharmacist-level barriers. Demographics included pharmacist and practice characteristics. The experience section of the survey asked respondents to report past experience with counseling patients with HIV infection, receipt of HIV-related continuing education, and familiarity with current guidelines regarding provision of PrEP. The willingness section contained the following header: “Truvada (emtricitabine/tenofovir) has been shown to reduce the risk of HIV infection by as much as 92% when used as pre-exposure prophylaxis (PrEP) in appropriate populations. The CDC has endorsed the use of emtricitabine/tenofovir for the prevention of HIV through guidelines released in 2014. The goal of this survey is to assess pharmacists’ willingness and perceived ability to dispense PrEP and monitor patients during the use of PrEP.” Respondents were then asked to rate how likely they would be to provide PrEP to clients at risk for HIV infection, after completion of training, and via participation in a collaborative practice agreement. In order to identify target areas for education, respondents were asked to rate their comfort completing PrEP-related tasks such as HIV risk assessment, point of care laboratory testing, and medication counseling for patients. Finally, respondents were asked to identify their concerns related to PrEP use in order to identify potential barriers to the implementation of pharmacy-based PrEP services. All items in the willingness and perceived abilities sections were measured on a Likert scale with the exception of a free-text answer item within each of the broader categories. Multiple responses were turned off in Survey Monkey to prevent respondents from submitting duplicate responses. All questions in the survey were optional and there was no time limit for completion.

### Statistical analyses

Pearson chi-square tests were used to determine if and to what extent demographics and familiarity were independently associated with respondent willingness to provide PrEP. Wilcoxon rank-sum tests were used to compare ages and years of experience between groups of respondents. Statistical analyses were conducted using SAS version 9.3 and P-values less than 0.05 were considered significant.

## Results

### Survey respondents

The survey invitation was e-mailed to 1,233 pharmacists in Nebraska and Iowa. There were 93 that failed delivery, resulting in 1,140 pharmacists who received the invitation. After the initial e-mail and one reminder, there were a total of 140 respondents, representing a response rate of 12.3%.

Overall, 54% of the respondents were female, 96% were white, 58% practiced in an urban setting, and the mean age was 45 years (See Table 1). Most respondents practiced in either academic (24%), independent (25%), or inpatient (32%) pharmacy settings. The majority of respondents (83%) reported having a PharmD or higher training related to the pharmaceutical sciences. A small proportion (9%) of respondents reported having specialty training in infectious diseases or practicing in an infectious diseases specialty.

**Table 1:** Demographics of responding pharmacists.

Demographics	N (Number of non-missing responses)	Mean (Range) or n (%)
**Age**	138	45.3 (26–73)
**Years in Practice**	138	19 (1–50)
**Female**	139	75 (54%)
**Race**	140	
**White/Caucasian**		135 (96%)
**Black or African American**		1 (1%)
**Other**		4 (3%)
**Setting of Pharmacy**	138	
**Urban**		80 (58%)
**Suburban**		17 (12%)
**Rural**		41 (30%)
**Type of Pharmacy**	134	
**Academic**		32 (24%)
**Ambulatory Care**		7 (5%)
**Inpatient Care**		43 (32%)
**Independent**		34 (25%)
**Chain**		10 (7%)
**Other**		8 (6%)
**Highest Level of Education**	140	
**Bachelor of Science**		25 (18%)
**Pharm D**		73 (52%)
**PGY1 or equivalent**		26 (19%)
**PGY2 or equivalent**		15 (11%)
**Fellowship**		1 (1%)
**Specialty training in infectious diseases**	140	12 (9%)

### Familiarity/Experience

Less than half of respondents were familiar with the use of PrEP (42%) or the CDC guidelines for its use (25%). Only 12% cited experience counseling patients on antiretrovirals specific for PrEP use, however 36% had counseled patients on antiretroviral therapy for treatment of HIV disease. Compared to those familiar with PrEP, respondents who were unfamiliar with the use of PrEP were older (47 vs. 42 years, p = 0.014) and had more experience (22 vs. 16 years, p = 0.004).

### Pharmacists’ willingness to provide PrEP services

Just over half (54%) of respondents indicated that they were fairly or very likely to provide PrEP services as part of a collaborative practice agreement and after additional training. While familiarity with PrEP use (p = .346) or CDC guidelines (p = .378) did not affect respondents’ willingness to provide PrEP, respondents were more likely to express an interest in providing PrEP services if they had prior experience counseling HIV-infected patients on antiretroviral therapy (OR 2.43, p = 0.023) or HIV-negative patients receiving PrEP (OR 4.67, p = 0.013), and if they had recently completed HIV-related continuing education (OR 2.77, p = 0.032) (See Table 2).

**Table 2:** Associations between familiarity/experience and likelihood of providing PrEP.

How likely do you think you would be to provide PrEP services to clients at risk for HIV after completion of additional training and participation in a collaborative practice agreement?					
	N	Somewhat, A little, or Not at all likely	Very or Fairly likely	Odds Ratio (95% CI)	P-value
Yes, I have counseled HIV-infected patients receiving antiretroviral therapy. n (%)	123	14 (25)	30 (45)	2.43 (1.12, 5.27)	0.023
Yes, I have completed HIV-related continuing education in the past year. n (%)	123	7 (13)	19 (28)	2.77 (1.07, 7.19)	0.032
Yes, I am familiar with the use of tenofovir/emtricitabine as pre-exposure prophylaxis (PrEP) for the prevention of HIV. n (%)	123	22 (39)	32 (48)	1.41 (0.69, 2.90)	0.346
Yes, I am aware of the current CDC guidelines for PrEP use. n (%)	123	12 (21)	19 (28)	1.45 (0.63, 3.33)	0.378
Yes, I have counseled patients on antiretroviral therapy for PrEP use. n (%)	123	3 (5)	14 (21)	4.67 (1.27, 17.19)	0.013
Yes, I have cared for at least one HIV-infected patient in the past year as part of my practice? n (%)	113	24 (46)	42 (69)	2.58 (1.20, 5.56)	0.015

Pharmacists were generally comfortable completing clinical tasks related to PrEP with greater than 80% reporting they would be comfortable with assessing HIV risk, performing and counseling on HIV testing, and providing medication counseling on PrEP use (See Table 3). However, nearly 30% expressed that they would not be comfortable performing urine-based pregnancy and STD testing.

**Table 3:** Pharmacists’ comfort with completing tasks related to PrEP.

Would you be comfortable completing each of the following tasks?	N	Yes, with my current knowledge and skill set n (%)	Yes, with some additional training n (%)	No, not even with additional training n (%)
Obtaining a medical history	123	87 (71)	34 (28)	2 (2)
Performing point of care serum creatinine testing	123	14 (11)	92 (75)	17 (14)
Collecting urine specimens for pregnancy and STD testing	123	17 (14)	70 (57)	36 (29)
Asking patients to self-collect oral and rectal swabs for STD testing	123	19 (15)	81 (66)	23 (19)
Assessing risk of HIV infection	122	24 (20)	87 (71)	11 (9)
Performing and interpreting point of care HIV testing	121	12 (10)	89 (74)	20 (17)
Counseling patients on their HIV testing results	123	15 (12)	93 (76)	15 (12)
Providing medication counseling on the use of PrEP for HIV	123	31 (25)	87 (71)	5 (4)

All associations (or lack thereof) noted above between measures of pharmacist’s familiarity and experiences and willingness to provide PrEP remained significant or not significant (respectively) in the context of multivariable models adjusting for years of practice, pharmacist’s sex, pharmacy setting (rural/suburban/urban), pharmacy type (academic vs. other), whether the pharmacist had a PharmD, and whether the pharmacist had received specialty training in infectious diseases (results not shown).

### Midwestern pharmacists’ concerns regarding the provision of PrEP services

**Fig 1:**
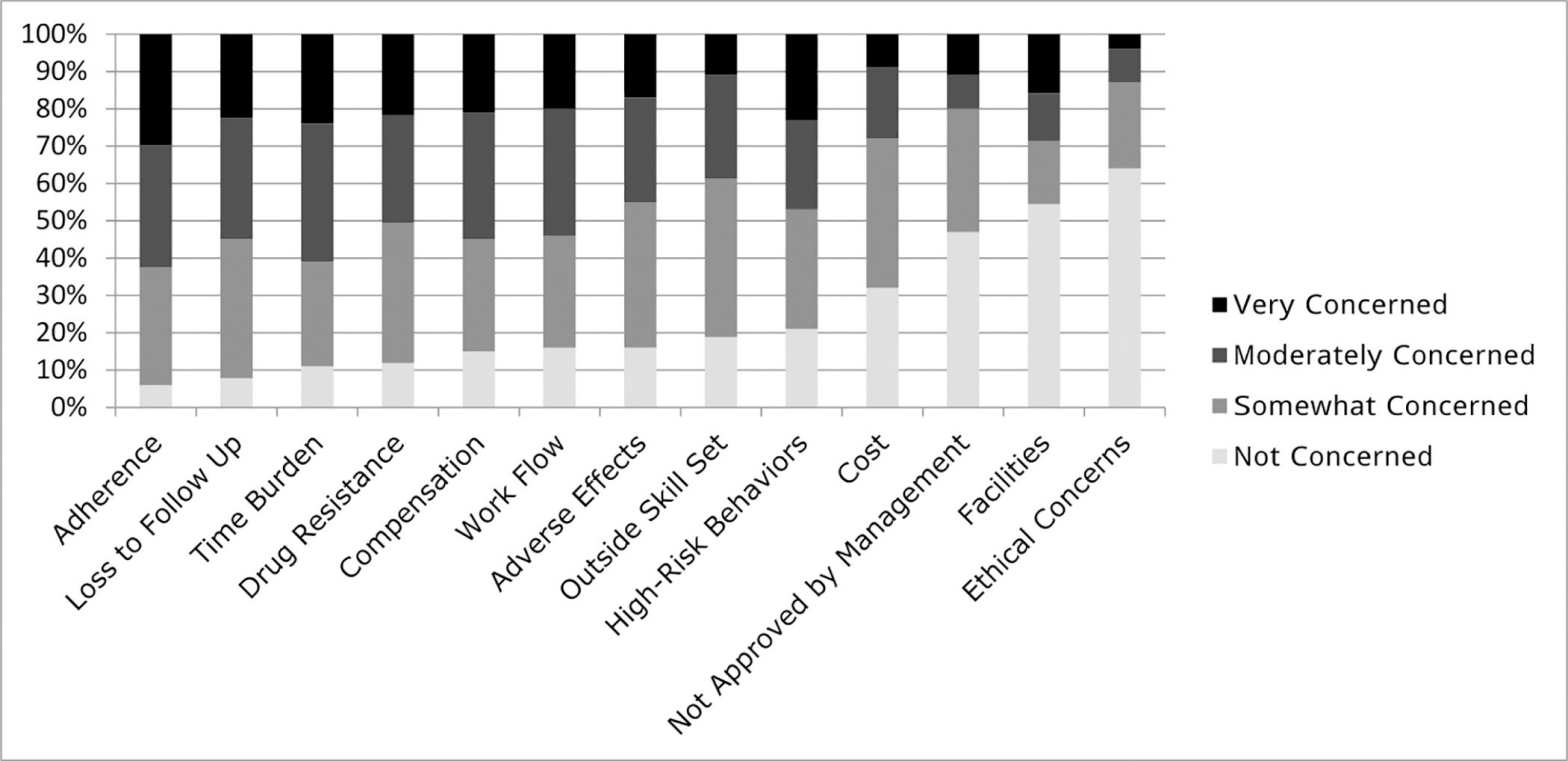
Pharmacist concerns related to PrEP. Pharmacists rated their PrEP-related concerns on a Likert scale including not concerned, somewhat concerned, moderately concerned, and very concerned. Responses are arranged from left to right according to increasing percentage of “not concerned” responses.

Respondents were “moderately concerned” or “very concerned” about the following issues: time burden (61%), inadequate compensation for services (55%), outside skill set (39%), patient adherence to therapy (63%), loss to follow-up (56%), and promotion of antiretroviral drug resistance (51%) (Fig 1). Only 13% of respondents expressed ethical concerns related to PrEP. Optional free-text entries for pharmacist-related concerns further characterized some of these ethical concerns, with one pharmacist identifying a concern about “encouraging unsafe and immoral behavior,” and another asking, “How much money would you spend on abstinence counseling?” Another pharmacist expressed concern regarding acceptance by the medical community, stating “Pharmacists need to be supported by the medical community as a point of care provider or this service will never be taken seriously and the stats will not reflect the true success of the program.”

Regarding tasks related to dispensing PrEP and monitoring patients on its use, 84% of pharmacists felt comfortable performing and interpreting point of care HIV testing and 88% felt comfortable counseling patients on their results (see Table 3). Free-text entries in the survey related to PrEP tasks included “I would never want to draw somebody’s blood or analyze their body fluids” and “I’d rather not touch blood, especially in patients with HIV.”

## Discussion

The relatively slow uptake of PrEP in the Upper Midwest [4] combined with the long distance between PrEP providers has sparked interest in finding innovative solutions to reach those at risk for HIV infection. Pharmacists have the knowledge and skill set necessary to perform the key components of PrEP delivery, including HIV screening, medication and adherence counseling, and may be an excellent resource for those in need of PrEP services [11]. This study sought to characterize pharmacists’ familiarity, experience, and willingness to fill this role. Since provision of PrEP requires thorough clinical evaluation as well as close monitoring and follow up, it is of utmost importance that careful consideration of these issues be part of the implementation of any PrEP model. In the case of pharmacist-led PrEP in the setting of a collaborative practice agreement, it is the pharmacists themselves who must be willing and able to implement an effective PrEP protocol within their clinical practice. Our data demonstrate that although pharmacists in Nebraska and Iowa have limited familiarity with PrEP and the CDC guidelines related to PrEP, the majority are willing to participate in the provision of PrEP.

Our findings were similar to those of Yoong and colleagues who also found that familiarity with the concept of PrEP was not significantly associated with support for PrEP in their survey of Canadian pharmacists [18]. While Young and colleagues only included pharmacists with HIV experience in their survey, we included all pharmacists and found that respondents with PrEP- or HIV-related experience as part of their education or clinical practice were most willing to participate in the provision of PrEP. Therefore, providing educational experiences and clinical training to pharmacists in these areas during the implementation of a pharmacist-led PrEP model would likely lead to further buy-in from pharmacists and increased longitudinal success.

Pharmacists’ clinical concerns related to PrEP are similar to those that have been previously reported by other medical providers: adherence, loss to follow-up, and drug resistance [7, 8]. Equally important, however, are issues related to pharmacy infrastructure: time burden, compensation, workflow, cost, and approval by management. Some of these items (e.g. cost) have been identified as potential barriers by surveys of pharmacists in Florida and Canada as well, and these will require careful consideration as we identify and partner with pharmacists and pharmacies to provide PrEP in a way that is sustainable in the long term [17, 18].

Although the majority of respondents did not express any ethical concerns related to pharmacist-led PrEP delivery, some respondents took the time to express their concerns in the optional free-text portion of the survey as described earlier. While the pharmacy has the potential to remove much of the stigma associated with PrEP since it is a neutral venue (as compared to an HIV clinic, for example), we must recognize that not all pharmacists will support the use of PrEP. Furthermore, it will be important to ensure that messages that alleviate pharmacists’ concerns about potential unintended harms associated with PrEP use are included in educational interventions.

There are some limitations to our study that warrant further discussion. The response rate was relatively low at 12.3%. Although this is similar to a similar survey of pharmacists that was administered electronically, the low response rate limits the extent to which our findings can be generalized [17]. Additionally, the optional nature of the survey means it is subject to non-response bias as pharmacists who are not interested in HIV disease or PrEP-services may have been less likely to complete the survey. Finally, the survey was anonymous so we were not able to determine whether or not there were any significant differences between respondents and non-respondents.

Fifty eight percent of respondents reported working in an urban setting and this is overall representative of pharmacists working in Nebraska and Iowa [20]. Thirty two percent of respondents reported practicing in an inpatient pharmacy setting and 24% reported practicing in an academic setting. Although this could be viewed as a limitation, academic medical centers and inpatient settings are often opportune settings in which to provide HIV screening and referrals for prevention services. Therefore, information from pharmacists who practice in these settings is valuable and contributed to breadth of pharmacy practice settings represented in this study. Additionally, the correlation between HIV experience and willingness to provide PrEP remained significant when utilizing multivariate analyses to adjust for pharmacy location and type.

By providing PrEP-focused continuing education and acknowledging the potential barriers to pharmacist-led PrEP identified in our study, a pharmacist-led PrEP model could prove to be a valuable avenue for the scale-up of PrEP that is needed to have an appreciable impact on the HIV epidemic. This model could have an especially significant impact in regions where access to a physician with the time and resources necessary to follow the frequent monitoring schedule required for PrEP is not available within a reasonable distance. While the majority of the states in the United States permit some form of pharmacist-presciber collaborative practice authority, not all states’ laws and regulations support the implementation of CPAs [16]. This presents a potential policy opportunity as revision of the laws governing CPAs in those states would allow for more widespread implementation of pharmacist and physician collaborations to provide PrEP.

## Supporting information

S1 Appendix Online survey. (PDF)

S1 Dataset De-identified dataset. (XLSX)
