# Supplementary material for: Midwest pharmacists’ familiarity, experience, and willingness to provide pre-exposure prophylaxis (PrEP) for HIV
Source: PLoS One. 2018 Nov 14;13(11):e0207372. doi: 10.1371/journal.pone.0207372 (PMC6235377; doi:10.1371/journal.pone.0207372)
Supplement: S1 Appendix — (PDF) [file pone.0207372.s001.pdf]

S1 Appendix

University of Nebraska Medical Center

Pharmacist experience and beliefs regarding HIV pre-exposure prophylaxis (PrEP)

Background and Informed Consent

1. **INTRODUCTION**

You are being asked to participate in a study being conducted by a research team from the University of Nebraska Medical Center. Before agreeing to participate in this study, it is important that you read and understand the following explanation of the study. You are free to accept or decline participation in the study without consequence.

PURPOSE OF THE STUDY

This questionnaire has been designed to assess interest in as well as barriers and facilitators to the implementation of a pharmacist-led HIV prevention program.

WHO IS ELIGIBLE

As a pharmacist practicing in Nebraska or Iowa, you are eligible to participate.

STUDY PROCEDURES

• Participation in this study is voluntary. If you agree to participate, you will be asked to fill out this online questionnaire.
• This survey is confidential. Please do not include your name or any other identifying information other than what is asked in the survey.
• This survey takes about 10 minutes to complete.
• You have the option to not answer any questions which you find confusing, uncomfortable or choose to omit for any reason.
• You also have the option to withdraw your consent by not completing the questionnaire if you so wish for any reason.
• Once your responses have been submitted you will not be able to withdraw from the study.

CONFIDENTIALITY

All information obtained during the study will be held in strict confidence. Completed surveys will be securely stored until study completion, after which the surveys will be securely destroyed. The results of this study may be presented by the study team at conferences, seminars or other public forums, and published in journals.

POTENTIAL RISKS

There is a very slight risk of loss of confidentiality in all email, downloading, and internet transactions.

POTENTIAL BENEFITS

Benefits to you from participating in this study may include gaining some knowledge of pre-exposure prophylaxis (PrEP) and issues surrounding its use.

INFORMED CONSENT

By clicking the box below, you are agreeing that you have read and understood the informed consent form and agree to participate in the study. If you would like further information about this study, you may contact Dr. Sara Bares, Principal Investigator, at 402-559-2666 during business hours. This study has been approved by the Institutional Review Board at the University of Nebraska Medical Center (UNMC IRB # 258-16-EX).

○ I have read and understood the consent form and agree to participate in the study

University of Nebraska Medical Center

Pharmacist experience and beliefs regarding HIV pre-exposure prophylaxis (PrEP)

Demographics

2. City, State of Practice

[ ]

3. I am practicing in a state(s) that allows collaborative practice agreements:

○ Agree

○ Disagree

○ I don't know

4. Year of birth

[ ]

5. Years in practice (Please round to the nearest whole year.)

[ ]

6. Sex

○ Male

○ Female

2

7. What is your ethnicity? (Please select all that apply.)

- [ ] American Indian or Alaskan Native
- [ ] Asian or Pacific Islander
- [ ] Black or African American
- [ ] Hispanic or Latino
- [ ] White / Caucasian
- [ ] Prefer not to answer
- [ ] Other (please specify)

8. Type of pharmacy (Please select best fit)

- ○ Independent
- ○ Chain
- ○ Mass merchandiser
- ○ Supermarket
- ○ Industry
- ○ Academic
- ○ Ambulatory care
- ○ Inpatient care

9. Setting of pharmacy (Please select best fit)

- ○ Urban
- ○ Suburban
- ○ Rural

10. What is your highest level of education related to the pharmaceutical sciences?

- ○ Bachelor of Science
- ○ PharmD
- ○ PGY1 or equivalent
- ○ PGY2 or equivalent
- ○ Fellowship

11. I have specialty training in infectious diseases or am currently practicing in an infectious diseases specialty as part of my practice:

- ○ Agree
- ○ Disagree

University of Nebraska Medical Center

Pharmacist experience and beliefs regarding HIV pre-exposure prophylaxis (PrEP)

Experience

12. Select all that apply:

- ☐ I have counseled HIV-infected patients receiving antiretroviral therapy.
- ☐ I have completed HIV-related continuing education in the past year.
- ☐ I am familiar with the use of Truvada (tenofovir/emtricitabine) as pre-exposure prophylaxis (PrEP) for the prevention of HIV.
- ☐ I am aware of the current CDC guidelines for PrEP use.
- ☐ I have counseled patients on antiretroviral therapy for PrEP use

13. How many HIV-infected patients have you cared for in the past year as part of your practice?

University of Nebraska Medical Center

Pharmacist experience and beliefs regarding HIV pre-exposure prophylaxis (PrEP)

Beliefs

Truvada (emtricitabine/tenofovir) has been shown to reduce the risk of HIV infection by as much as 92% when used as pre-exposure prophylaxis (PrEP) in appropriate populations. The CDC has endorsed the use of emtricitabine/tenofovir for the prevention of HIV through guidelines released in 2014. The goal of this survey is to assess pharmacists' willingness and perceived ability to dispense

PrEP and monitor patients during the use of PrEP.

14. How likely do you think you would be to provide PrEP services to clients at risk for HIV after completion of additional training and participation in a collaborative practice agreement?

- ◯ Very likely
- ◯ Fairly likely
- ◯ Somewhat likely
- ◯ A little likely
- ◯ Not at all likely

15. Would you be comfortable completing each of the following tasks?

	Yes, with my current knowledge and skill set	Yes, with some additional training	No, not even with additional training
Obtaining a medical history	◯	◯	◯
Performing point of care serum creatinine testing	◯	◯	◯
Collecting urine specimens for pregnancy and STD testing	◯	◯	◯
Asking patients to self-collect oral and rectal swabs for STD testing	◯	◯	◯
Assessing risk of HIV infection	◯	◯	◯
Performing and interpreting point of care HIV testing	◯	◯	◯
Counseling patients on their HIV testing results	◯	◯	◯
Providing medication counseling on the use of PrEP for HIV	◯	◯	◯

5

16. How concerned would you be with...

	Not concerned	Somewhat concerned	Moderately concerned	Very concerned
Inadequate compensation for services	◯	◯	◯	◯
Time burden	◯	◯	◯	◯
Disruption of work flow	◯	◯	◯	◯
Outside skill set	◯	◯	◯	◯
Ethical concerns	◯	◯	◯	◯
My supervisors and/or management would not support this	◯	◯	◯	◯
Cost-effectiveness of PrEP	◯	◯	◯	◯
My pharmacy does not have a private space for counseling	◯	◯	◯	◯

Please share any other pharmacist-related concerns you may have:

17. How concerned would you be with...

	Not concerned	Somewhat concerned	Moderately concerned	Very concerned
Adherence to medication	◯	◯	◯	◯
Encouraging high-risk sexual practices	◯	◯	◯	◯
Promoting drug resistence	◯	◯	◯	◯
Adverse effects	◯	◯	◯	◯
Loss to follow-up	◯	◯	◯	◯

Please share any other patient-related concerns you may have:

Thank you for completing this survey. Your input is very much appreciated!
